# Survey of health literacy level and related influencing factors in military college students in Chongqing, China: A cross-sectional analysis

**DOI:** 10.1371/journal.pone.0177776

**Published:** 2017-05-17

**Authors:** Honghui Rong, Xin Cheng, Jose M. Garcia, Ling Zhang, Lu Lu, Jian Fang, Mingshan Le, Peng Hu, Xinlu Dong, Junli Yang, Ya Wang, Ting Luo, Jun Liu, Ji-an Chen

**Affiliations:** 1 Department of Health Education, Third Military Medical University, Chongqing, China; 2 GRECC, VA Puget Sound Health Care System and University of Washington, Seattle, WA, United States of America; 3 Department of Ambulant Clinic, Institute of Communication of PLA, Chongqing, China; 4 Department of Ambulant Clinic, Institute of Logistics Engineering of PLA, Chongqing, China; Oulun Yliopisto, FINLAND

## Abstract

Health literacy (HL) has become an important public health issue and is receiving growing attention. However, the HL levels of military college students in China have never been analyzed. This study aimed to investigate the HL and related associate factors in military college students in Chongqing, China. Data was obtained with the “Chinese Citizen Health Literacy Questionnaire (2012 edition)” from 3183 military college students aged 16–28 years at Chongqing in December 2015. A total score of ≥80 points determined adequate HL, and HL level was defined as the proportion of students who had adequate HL out of the total number of participants. Multiple logistic regression analysis with a stepwise forward likelihood ratio (LR) method was used to determine the effects of sociodemographic characteristics, health-related behaviors, and family-related factors on HL level. The mean score of HL was 68.56, and the HL level of military college students was 21.05%; the overall knowledge rate was 71.33%. The independent factors that were associated with HL level were years in college, educational system, time playing online games, annual household income and father's education level. Senior (odds ratio [OR] = 1.229, 95% confidence interval [CI] 1.018∼1.484), undergraduate (OR = 1.509, 95% CI 1.151∼1.978), time played games more than 5 hours each week (OR = 0.638, 95% CI 0.486∼0.837), annual household incomes more than 50,000 yuan (OR = 1.231, 95% CI 1.027∼1.476) and father's education level (high school: OR = 2.327, 95% CI 1.186∼4.565; university: OR = 2.450, 95% CI 1.244∼4.825), were independently associated with higher HL level. HL levels of military college students in Chongqing need to be improved across the board. Our data suggests that special emphasis should be placed on students in junior and those in the specialist educational system. School departments may also benefit from incorporating health literacy into their curricula and helping students manage the time they spend playing online games.

## Introduction

Health literacy (HL) is the degree to which an individual has the capacity to obtain, process, and understand basic health information and services to make appropriate health decisions [1]. Health literacy has become an important public health concern and has received growing attention in recent years because of its critical role in health education and promotion [2,3]. As a variable associated with health outcomes, health literacy is an important component of health quality, health behaviors, and access to health care [4–7]. Many empirical studies have identified significant associations between inadequate health literacy and poor health outcomes. For example, low health literacy level is associated with a reduced likelihood of understanding diagnoses, treatment plans [8], and prescription drug label instructions [9]. Emergency department patients with poor health literacy might be subject to an increased risk of hospitalization [10]. Evidence suggests that people with low health literacy tend to have a reduced understanding of the necessities and benefits of using preventive care services, including cancer screening tests [11]. In addition, the high prevalence of low health literacy has been found to be associated with certain deteriorating cognitive abilities, such as memory and verbal fluency, among the elderly [12].

The concept of health literacy was introduced in China in 2005 by the Chinese government through a manual entitled “Basic Knowledge and Skills of People’s Health Literacy” [13]. In 2008, the Chinese Ministry of Health developed a corresponding measure called “HL for Chinese Citizens—Basic Knowledge and Skills”. This 66-item questionnaire addresses three components: basic knowledge and beliefs, knowledge and attitudes toward health-related behaviors and lifestyles, and basic skills [14]. Using this questionnaire, a nationally representative survey of 79,542 people showed that only 6.48% of participants had adequate HL; there were urban–rural and regional differentials, with lower percentages of adequate HL in rural and western residents [15]. Based on this survey, other studies have been conducted to examine the relationship between health literacy and related risk factors [16,17] as well as health status in an elderly minority group [18]. The concept of HL is sensitive to context and culture, and its meaning can differ between cultures/countries or racial/ethnic groups [19]. Other studies using self-designed health literacy scales have investigated the relationship between health literacy and health education in elementary and middle school students [20], infectious disease patients [21] and regarding ethnic disparities in health-related quality of life [22]. Many studies have focused on the prevalence and distribution of HL in different population groups (including the elderly, children, doctors, college students, etc); however, few have examined the HL of military college students.

Military college students are not only an important component of military systems but are also the future senior professionals of the army. Like students in civilian universities, military college students are affected by physiological, psychological and social factors, but they also have the special characteristics of military academy because they live in a special campus environment and accept strict militarization management [23]. They assume the dual mission of college students and military personnel with the accompanying pressures of civilian colleges and universities, such as attending classes and taking exams, and also their military duties. This may place an extra burden on their HL and health status. To date, limited data is available regarding the relationship between health literacy and health-related behaviors and health status in military college students, underscoring the importance of studying these and possible interventions to implement in this unique and important population.

The aim of this study was to evaluate the relationship between HL and health-related behaviors and background variables in military college students. The objectives of this study are to: 1) investigate the health literacy status of military college students in Chongqing, China, and 2) analyze the risk factors that affect the health literacy of this population. If risk factors can be identified, health education and promotion interventions may be used to target high-risk populations to improve their health literacy. The study findings may provide evidence to support the development of related health policies in China’s army.

## Materials and methods

### Participants

**Fig 1:**
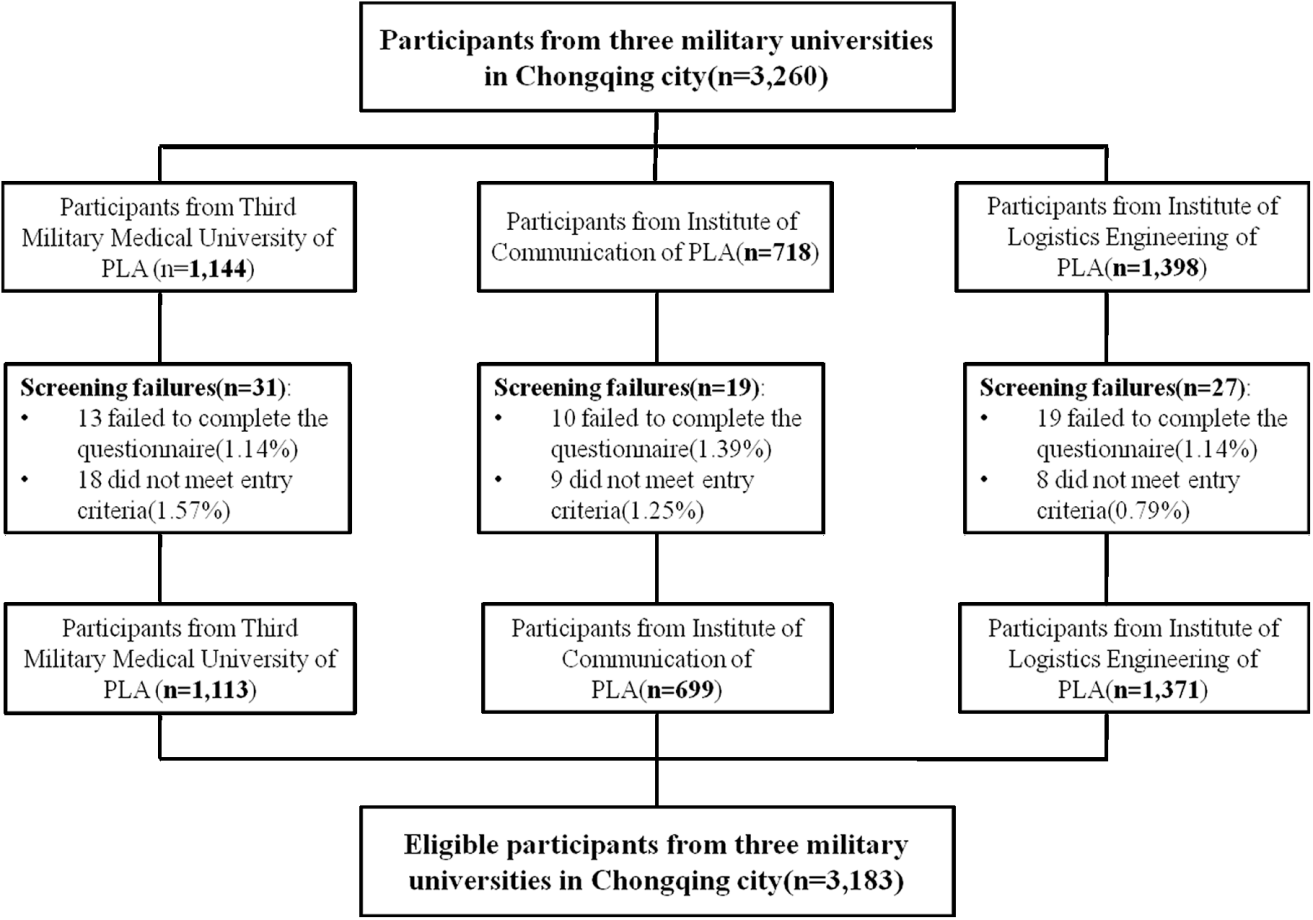
A cluster random sampling method was adopted to identify participants in this study. 3,260 participants came from three military universities in Chongqing city China, including Third Military Medical University of PLA 1,144, Institute of Communication of PLA 718, and Institute of Logistics Engineering of PLA 1,398, respectively. After exclusion of screening failures, the eligible participants were 3,183, including Third Military Medical University of PLA 1,113, Institute of Communication of PLA 699, and Institute of Logistics Engineering of PLA 1,371, respectively.

This was a cross-sectional study. The target participants were military college students currently attending one of three colleges (Third Military Medical University of PLA, Institute of Communication of PLA, Institute of Logistics Engineering of PLA) in Chongqing city, China, and were volunteers from all levels. The educational system in China was as follows: primary or elementary (grades 1–5), high school (years 6–12), college or University with two different systems: a 3 year path or “specialist” and a 4 year path or “undergraduate". Also, college can be divided in ‘junior’ for the first two years or ‘senior’ for subsequent years. The survey was conducted from November to December in 2015. A cluster random sampling method was adopted to identify participants (Fig 1). Participants who attended college were included Subjects that failed to complete the questionnaire were excluded. A total of 3410 questionnaires were issued and 3183 of them were completed. Hence, the response rate of questionnaire was 93.34%. The purpose of the survey was explained to participants, and they were asked to give verbal informed consent. Before the start of the interview, participants were asked to provide their sociodemographic information. Written informed consent was obtained from every student before providing them with the questionnaires, The study was approved by the Ethics Review Board of the Third Military Medical University.

### Instruments

The two instruments used in this study were the Chinese Citizen Health Literacy Questionnaire (China Health Education Centre, 2012) [24] and a Scale of General Status, which included three sections: demographic characteristics of the individual student (sex, age, ethnicity, school, years in college, educational system, city of residence before entering university), students' health behaviors (smoking, alcohol use, playing of online games weekly), family situation and parent-related information (annual household income, parents' education level and occupation). For these indicators, years in college was divided into junior (years 1–2) or senior (years 3–4), a smoker was defined as someone who had smoked at least one cigarette per day for more than six months or quitting less than six months prior to recruitment [25]; a drinker was defined as someone who drinks alcohol at least once a week for more than six months [25].

This questionnaire was designed by experts in public health, health education and promotion, and clinical medicine using the Delphi method. Details of the development procedure were described in a previous paper [24], and our pre-experiment with 300 people showed that the overall Cronbach’s alpha was 0.922; Cronbach’s alpha of the three dimensions were as follows: 0.853 (knowledge and attitude, 38 items); 0.717 (knowledge of and attitudes toward health-related behaviors and lifestyles, 22 items); and 0.820 (skills, 20 items).The two-parameter logistic model fitted the data well (P>0.05)[14]. Classical test theory (Cronbach’s alpha, split-half coefficient, and factor analysis) and modern test theory (IRT) were used in validating the scale and Shen et al [14] found that the 2012 scale of health literacy meets psychometric standards. The questionnaire includes 80 items. Based on the findings by Li [24] and Shen[14], health literacy was divided into three dimensions:(1) knowledge and attitudes (KAA); (2) knowledge and attitudes toward health-related behavior and lifestyle (BAL); and (3) health-related skills (HRS) or six domains: scientific views of health (SVH); infectious diseases (ID); chronic diseases (CD); safety and first aid (SAFA); medical care (MC); and health information (HI). There are four types of questions in the scale: true-or-false; single-answer (only one correct answer in a multiple-choice question); multiple-answer (more than one correct answer in a multiple-choice question); and situation questions. With multiple-answer questions, the correct response had to contain all of the correct answers and none of the wrong ones. Situation questions were asked following a paragraph of instruction or medical information. A total score of 80 points and above was determined to indicate adequate health literacy [14], and health literacy level was defined as the proportion of participants who had adequate health literacy out of the total number. The total KAA, BAL, and HRS scores are 47, 28, and 25 points, respectively, and those of the SVH, ID, CD, SAFA, MC and HI are 17, 13, 20, 19, 18, and 13 points, respectively [14]. Each score that reached 80 percent and above was determined to indicate health literacy in that one aspect, and the ratio of the number literate in each category to the corresponding total number of participants represented the literacy level of that [14]. HL knowledge rate was defined as the ratio of correct topics that the participants answered to the percentage of incorrect topics; the knowledge rate of the three dimensions and six aspects of HL were calculated similarly. The knowledge rate (%) was calculated using the following formula: Total number of correct answers/(Total number of questions for each questionnaire × Total number surveyed) × 100%.

### Statistical analyses

All data were entered in duplicate into an Epidata version 3.1 database, and data entry screens were used to revise the incorrect entries (i.e., input and logic errors). Data were analyzed with SPSS (SPSS18.0; Stat Soft Inc, Tulsa, USA). Measurement data were presented as Mean ± Std, and we performed descriptive statistics on the distributions of the three dimensions and six aspects of HL; additionally, one-way ANOVA was used to compare the differences between them. Enumeration data were expressed as the rate or constituent ratio, while the literacy level and knowledge rate of the three dimensions and six aspects of HL were compared with chi-square test. Categorical variables were presented as the frequency percentage, and intergroup comparisons were performed using chi-square test. Multiple logistic regression was used to adjust for the risk factors associated with HL, and we used other covariates as well, including adjusting for major (Non-medical profession = "0"; medical profession = "1"); years in college (junior = "0"; senior = "1"); educational system (specialist = "0"; undergraduate = "1"); residence prior to the study (rural = "0"; urban = "1"); time playing online games (<5 hours in a week = "0"; ≥5 hours in a week = "1"); household annual income (<50,000 yuan = "0; ≥ 50,000 yuan = "1") (the price of 50,000 yuan is the median chinese income); and parents' education level (primary and less = "1"; high school = "2"; university = "3"; postgraduate = "4"). The odds ratios (OR) and 95% confidence intervals (95%CI) were determined for all variables. The HL equation was established using a multiple logistic regression model (a_in_ = 0.05, a_out_ = 0.10) with a stepwise forward (LR) method. The results of all hypothesis tests with p-values<0.05 (two-sided) were considered to be statistically significant.

## Results

### Average score, literacy level and knowledge rate of the three dimensions and six aspects of HL

A total of 3183 military college students aged 16–28 years were enrolled in the study, and the effective response rate was 93.34%. As shown in Table 1, the mean HL score was 68.56 (Std = 14.66). The three dimensions of HL in order of decreasing average score were KAA (72.68), BAL (66.73), and HRS (62.85). The six aspects of HL in order of decreasing average score were SVH (78.12), SAFA (75.30), CD (70.08), HI (67.76), ID (60.69), and MC (56.97).

**Table 1:** Average score, literacy level and knowledge rate of the three dimensions and six aspects of HL.

Parameters	Mean±Std^a^	Invalid	Valid	Level(%)	Correct	Incorrect	Knowledge rate(%)
**Three Dimensions**							
KAA	72.68 ± 14.17	2081	1102	34.62	91351	29603	75.53
BAL	66.73 ± 15.37	2344	839	26.36	49038	20988	70.03
HRS	62.85 ± 20.32	2463	720	22.62	41235	22425	64.77
**Six Aspects**							
SVH	78.12 ± 15.83	1480	1703	53.5	38623	9122	80.89
ID	60.69 ± 20.09	2279	904	28.4	21161	10669	66.48
CD	70.08 ± 15.73	2053	1130	35.5	35560	12185	74.48
SAFA	75.30 ± 18.78	1199	1984	62.33	34865	12880	73.02
MC	56.97 ± 17.65	2805	378	11.88	28590	19155	59.88
HI	67.76 ± 22.16	1758	1425	44.77	22825	9005	71.71
**HL**	68.56 ± 14.66	2513	670	21.05	181624	73016	71.33

KAA (knowledge and attitudes); BAL (knowledge and attitudes toward health-related behavior and lifestyle); HRS (health-related skills); SVH (scientific views of health); ID (infectious diseases); CD (chronic diseases); SAFA (safety and first aid); MC (medical care); HI (health information)

^a^: all scores have been standardized.

Six hundred-seventy of the 3183 (21.05%) participants had an adequate HL level, while the literacy levels of the KAA, BAL, HRS subscales were 34.62%, 26.36%, and 22.62%, respectively. Additionally, the literacy levels for the six aspects of HL in descending order were 62.33%, 53.50%, 44.77%, 35.50%, 28.40%, and 11.88% for SAFA, SVH, HI, CD, ID, and MC, respectively.

The total number of items answered by the 3183 participants was 254,640 (3183*80), and the overall knowledge rate was 71.33%. The rate of knowledge in the KAA, BAL, and HRS dimensions was 75.53%, 70.03% and 64.77%, respectively. Additionally, for the six aspects of HL, the rates of knowledge in descending order were 80.89%, 74.48%, 73.02%, 71.71%, 66.48%, and 59.88% for the SVH, CD, SAFA, HI, ID, MC subscales, respectively.

### The association between HL level and sociodemographic characteristics, health-related behaviors, and family-related factors

Chi-square test was used to compare the differences in age, ethnicity, gender, major, years in college, education system, and prior residence. There were significant differences in HL levels as a function of years in college and education system (P<0.05), as shown in Table 2.

**Table 2:** Association between HL levels and sociodemographic characteristics, health-related behaviors, family-related factors of the 3183 military college students.

Parameters		Percentage (%)	Adequate HL (HL level %)	Chi-square^c^	P
**Sociodemographic characteristics**					
**Major**	Non-MP	2070(65.03)	418(20.19)	2.611	0.106
	MP^b^	1113(34.97)	252(22.64)		
**Gender**	Female	444 (13.95)	101(22.75)	0.896	0.344
	Male	2739(86.05)	569(20.77)		
**Age**	16∼20	1693(53.19)	340(20.08)	2.313	0.315
	21∼25	1391(43.70)	306(22.00)		
	26∼28	99 (3.11)	24 (24.24)		
**College**	IC	699(21.96)	118(16.88)	9.577	0.008
	ILE	1371(43.07)	300(21.88)		
	TMMU	1113(34.97)	252(22.64)		
**Ethnicity**	Han	2948(92.62)	624(21.17)	0.332	0.564
	Other	235 (7.38)	46 (19.57)		
**Years in college**	Junior	2272(71.38)	452(19.89)	6.372	**0.012**
	Senior	911 (28.62)	218(23.93)		
**Educational system**	Specialist	491 (15.43)	77 (15.68)	10.063	**0.002**
	Undergraduate	2692(84.57)	593(22.03)		
**Residence**	Rural	907 (28.50)	184(20.29)	0.444	0.505
	Urban	2276(71.50)	486(21.35)		
**Health-related behaviors**					
**Smoking**	Non-smoking	2924(91.86)	622(21.27)	1.074	0.300
	Smoking	259 (8.14)	48 (18.53)		
**Alcohol use**	Has not used	3097(97.30)	651(21.02)	0.058	0.810
	Has used	86 (2.70)	19 (22.09)		
**Time playing online games**	<5 Hours	2735(85.93)	598(21.86)	7.774	**0.005**
	≥5 Hours	448 (14.07)	72 (16.07)		
**Family-related factors**					
**Annual household income**	< 50,000 yuan	1860(58.44)	365(19.62)	5.473	**0.019**
	≥ 50,000 yuan	1323(41.56)	305(23.05)		
**Father's education level**	Primary and less	394 (12.38)	61(15.48)	16.412	**0.001**
	High school	1815(57.02)	399(21.98)		
	University	878 (27.58)	200(22.78)		
	Postgraduate	96 (3.02)	10(10.42)		
**Mother's education level**	Primary and less	627 (19.70)	111(17.70)	11.764	**0.008**
	High school	1888(59.32)	421(22.30)		
	University	616 (19.35)	134(21.75)		
	Postgraduate	52 (1.63)	4 (7.69)		
**Father's occupation**	Teacher	142(4.46)	33 (**23.24**)	2.810	0.729
	Medical staff	75 (2.36)	16 (21.33)		
	Civil servant	901(28.31)	199(22.09)		
	Farmer	830(26.08)	160(19.28)		
	Worker	392(12.32)	80 (20.41)		
	Other	843(26.48)	182(21.59)		
**Mother’s occupation**	Teacher	196(6.16)	48 (**24.49**)	6.293	0.279
	Medical staff	100(3.14)	20 (20.00)		
	Civil servant	692(21.74)	145(20.95)		
	Farmer	949(29.81)	181(19.07)		
	Worker	326(10.24)	64 (19.63)		
	Other	920(28.90)	212(23.04)		

^b^ "MP" represents medical profession

^c^ Pearson Chi-Square.

HL level was significantly associated with time playing online games (P<0.05). The participants who played computer games for less than five hours a week had higher HL levels than those spending more than five hours, at 21.86% and 16.07%, respectively. Interestingly, no differences were found between HL level and smoking or drinking (P>0.05 for both).

HL levels were significantly associated with annual household income, father's education level, and mother's education level (P<0.05). Those whose annual household income was greater than 50,000 yuan had higher HL levels than those whose annual household income was less than 50,000 yuan (P<0.05). Moreover, the relationship between HL levels and parents' education level was significant; specifically, the HL level of students whose parents had a postgraduate education was the lowest. Interestingly, the HL levels of students whose parents were teachers were the highest for both mother and father’s occupation.

### Multiple logistic regression analysis of factors influencing HL level including sociodemographic characteristics, health-related behaviors, and family-related factors

Forward stepwise-wald method was used in multivariate logistic regression analysis. In omnibus tests of model coefficients, Chi-square = 48.013, p<0.001, so the overall model was significant. As shown in Table 3, individuals in senior were significantly more likely to have a higher HL level than those in junior. There were significant differences between specialist and undergraduate school systems. Students who played games more than 5 hours each week tended to have lower HL levels than those who played for less than 5 hours. Students with annual household incomes more than 50,000 yuan were likely to have a higher HL level. Compared to students whose father's education level was postgraduate, students whose father's education level was high school and university had significantly higher HL levels. Of these factors, years in college, educational system, annual household income, and father's education level except postgraduate education were positively correlated with health literacy level, while time playing online games was negative correlated with health literacy level.

**Table 3:** Multiple logistic regression analysis of factors influencing HL level with sociodemographic characteristics, health-related behaviors, and family-related factors.

Parameters		B	S.E.	Wald	P	OR	95%CI of OR
**Years in college**	Senior	0.207	0.096	4.618	0.032	1.229	(1.018∼1.484)
	Junior	reference					
**Educational system**	Undergraduate	0.411	0.138	8.855	0.003	1.509	(1.151∼1.978)
	Specialist	reference					
**Time playing online games**	≥5 Hours	-0.449	0.139	10.506	0.001	0.638	(0.486∼0.837)
	<5 Hours	reference					
**Annual household income**	≥ 50,000 yuan	0.208	0.092	5.072	0.024	1.231	(1.027∼1.476)
	< 50,000 yuan	reference					
**Father's education level**	Primary and less	0.453	0.369	1.506	0.220	1.573	(0.763∼3.241)
	High school	0.844	0.344	6.031	0.014	2.327	(1.186∼4.565)
	University	0.896	0.346	6.719	0.010	2.450	(1.244∼4.825)
	Postgraduate	reference					
**Constant**		-2.566	0.354	52.554	0.000	0.077	

## Discussion

Health literacy covers scientific literacy, education system, information technology, media literacy, existing health care infrastructure, health care system and other factors. Health literacy is an important factor in evaluating results of health promotion, quality of life and the overall health level of citizens, so governments and researchers need to be aware of this factor. The United States Government has proposed that improving health literacy is one of the key objectives of the White Paper on Health Policy [26]; In early 2008, China issued the "Chinese Citizens 'Health Literacy—Basic Knowledge and Skills (Trial)", which is the first government document to define citizens' health literacy in the world [27]. Clinical medicine and public health are the two main directions in the field of health literacy research [28]. In the clinical field, lower literacy results in poor communication quality, patient outcomes, and long-term health status of the patient [29]. Therefore, improving health literacy may help improve the relationship between doctors and patients, reduce medical disputes, improve compliance and promote rehabilitation. In the field of public health, low literacy hinders the improvement of individuals' health knowledge, awareness and the development of personal health skills, and restricts the effectiveness of health promotion activities [30]. Low HL was also found to be harmful to health status of communities and even the whole population in some public safety events [31]. The application of health literacy in the field of public health could guide healthcare workers to improve health promotion programs, and provide high-affinity, high-impact health information for people, assist people to master more operational health skills, and improve health literacy in the whole population. Health literacy is an important predictor of health status and low health literacy may lead to an increase in morbidity and prevalence of several diseases [32]. Although health literacy does not directly affect health status, it could do so indirectly by influencing knowledge of disease, healthy lifestyles and effective use of preventive health services. Health literacy may improve survival, save costs, and improve the health of hundreds of millions of people.

In 2008, the first national survey results showed that the health literacy level of Chinese citizens was 6.48% [15], and these levels were 8.80%, 9.48%, and 9.79% in 2012, 2013 and 2014, respectively [33–35], indicating an increase each year. In this study, the health literacy level of students in three military colleges in Chongqing was 21.05%, which was higher than the health literacy level of the general Chinese population in other studies. The levels of literacy in the three dimensions (KAA, BAL, HRS) and six aspects (SVH, ID, CD, SAFA, MC, HI) of HL were higher in our population than in a previous report when 15 to 24 years old Chinese residents were surveyed [36]. In addition to ID and MC, the health literacy levels of the other seven items were higher in military college students than in Chinese residents. This may be associated with the education level of these students, as the ability to analyze and understand health information improves with increases in education level [37–39].

In our study, the multivariate logistic regression analysis indicated that years in college, educational system, time playing online games, annual household income, father's education level, both high school and university, may affect the health literacy level of military college students. In terms of years in college (junior v. senior), this survey showed that senior students had a higher health literacy level than junior students (OR = 1.229 and 95% CI of 1.018 ∼ 1.484). Junior students' main task is the basis of professional knowledge, while senior students focus on practical exercises and military training. A possible explanation of this finding was that junior students had just finished their college entrance examinations, which required intense studies, and thus they perceived more severe pressure and had focused on learning scientific theory and cultural knowledge, neglecting their health. In contrast, senior students had adapted to college life, and thus their overall health literacy level and understanding of health concepts had improved with the accumulation of their knowledge [40,41].

The health literacy level of undergraduate students was 0.509 times higher than that of specialist students, which may be related to their high school-level knowledge base. In our study, the level of health literacy of undergraduate students is higher than that of specialist students, which is consistent with Yang et al [42]. It may be due to the ability of undergraduates to learn or accept knowledge independently. Generally, college students who have poor basic knowledge have more academic difficulties in university and experience more severe pressure; therefore, they may mainly be concerned with learning theoretical knowledge and have no time to attend practical operations and exercise, ultimately affecting their health literacy levels.

The military college students who played online games for more than 5 hours weekly had lower health literacy levels. The possible explanation for this finding is that the time they spent playing games reduced their learning and training opportunities, which delayed their health literacy training and promotion. Tang et al [43] showed that students with low health literacy are more likely to be addicted to the Internet than those with high health literacy. Besides, a total of 5400 university students were investigated with the original version of the Healthy Lifestyle Scale for University Students by two-stage stratified cluster sampling method and after the original scale was evaluated and revised according to the structural equation modeling, the reliability and construct validity of the revised version of Healthy Lifestyle Scale for University Students were tested, and the playing online game was incorporated into the questionnaire items [44].

This study showed that the health literacy level was higher in military college students whose annual household income was more than 50,000 yuan than in those with less than 50,000 yuan (OR = 1.231, 95%CI was 1.027∼1.476), which is consistent with the results of Liu et al [45]. This suggests that higher health literacy levels are associated with economic factors. One explanation could be that with improvements in family income level, family members have more energy to devote to their own health and that of their relatives. They place a greater emphasis on health and have the ability to provide better housing and living conditions, which positively improves overall health [46]. Another reason could be that the pressure they experienced in life was reduced because of their higher household income, and thus they were able to maintain their family members’ health. In addition, a higher household income also improves the quality of a person’s diet, which can improve health status.

Using postgraduate education as the reference, military college students whose father's educational level was high school or university had a higher health literacy level, which was consistent with the results of Liang et al [47]. Parents' education level influences children's health knowledge, attitude and behavior, as reported by Piko et al [48]. It is worth noting that in the univariate analysis, the health literacy level was the lowest for military college students whose parents had a postgraduate educational level (father (10.42%) and mother (7.69%)). A possible reason for this finding was that parents who have postgraduate education may engage in more time-consuming activities, so they dedicate less time to their children’s education. The health literacy levels were the second lowest for military college students whose fathers’ and mothers’ education levels were primary school and less. The health literacy level of military college students increased with improvements in parents' educational level, which indicated that parents' education may have some impact on students' health literacy. Taking together, the data suggest that having parents with a higher health literacy level could affect and promote the awareness of the importance of health literacy in their children, cultivate good habits and behavior, and promote adequate health literacy [49]. More studies are needed to confirm this and also to explore the reason why parental postgraduate education was associated with lower HL.

Although we would have expected that pursuing a medical profession would be predictive of higher health literacy levels, we did not find a significant relationship between profession and health literacy, which was inconsistent with Zhang et al [50]. Further studies would be needed to confirm this finding and explore its potential causes.

In addition, the health literacy level of military college students whose fathers and mothers were teachers was the highest (father (33, 23.24%), mother (48, 24.49%)) of the parental occupations, although the differences were not statistically significant. This suggests that parents who were teachers may pay more attention to the overall quality of their children's education. Li et al [51] analyzed the influencing factors of health literacy of college students in the city of Changsha and point out that health literacy level of the students whose parents are teachers is relatively high.

The “Chinese Citizen Health Literacy Questionnaire (2012 edition)” does have some limitations. As recently pointed out by Shen et al [14], the 2012 national health literacy scale was modified, and 64 items were selected based on classical test theory and item response theory. This survey was conducted from November to December in 2015, and at that time, the Chinese government had not updated the questionnaire, so we still used the 80 items questionnaire. It may not be valid to compare the health literacy level among different countries because of the difficulty of measuring health literacy. Each country has its own reference standard for health literacy. In China, a total score of 80 points and above indicated adequate health literacy, and health literacy level was defined as the proportion of participants who had adequate health literacy out of the total number. Other limitations in this study include: the cross-sectional study design that makes causal relationships undeterminable; the fact that the Chinese citizens' health literacy questionnaire was developed for civilians and it may not be suitable for military personnel; the focus of this study is military college students in Chongqing, and these results may not apply to other populations; As noted above, the test is time consuming: it usually took 30 min for an adult to complete and even longer for participants with limited literacy or other conditions. Nevertheless, this cross-sectional survey provided important evidence regarding health literacy and identified several risk factors in a population in China and the findings of this study added evidence into the limited literature on health literacy among military college students. In future studies, outcomes associated with a poor status of health literacy should be explored.

## Conclusions

This is, to our knowledge, the first population-based study to examine the HL of military college students in Chongqing, China. The general health literacy level of military college students in Chongqing in our study was high relative to the general population but still low (health literacy level of them was 21%). Years in college, educational system, time playing online games, annual household income and father's education level are predictors of health literacy in military college students. Specific measures that may be adopted in future strategies are needed. We propose that health education should be provided to military college students with a special focus on junior students and in the specialist educational system through a variety of means, including media, advertising material, and a series of health education lectures to improve the health literacy of military college students. Also, school departments should incorporate health literacy into their curricula and adopt effective measures to strengthen students’ development of a healthy lifestyle, in particular to manage the time students spend playing online games. Finally, parents should also actively promote health education, and be aware of their children's health awareness.

## Supporting information

S1 File Detailed analysis of each index. This excel file include three shifts: comparision about scores of HL, compaision about level of HL, p value summary.
(XLS)

S2 File The Scale of general status in original language. (DOC)

S3 File The Scale of general status in English. (DOC)
