# Supplementary material for: Survey of health literacy level and related influencing factors in military college students in Chongqing, China: A cross-sectional analysis
Source: PLoS One. 2017 May 17;12(5):e0177776. doi: 10.1371/journal.pone.0177776 (PMC5435342; doi:10.1371/journal.pone.0177776)
Supplement: S2 File — (DOC) [file pone.0177776.s002.doc]

军校学员基本信息调查表

**1.人口学特征**

1.1 性别： A.男 B.女

1.2 年龄： 岁

1.3 民族： A.汉族 B.其它

1.4 所在学校： ；

1.5 专业类别：A.医学 B.非医学

1.6 年级: A.低年级（大一及大二） B.高年级（大三及大四）

1.7 学制: A.专科 B.本科

1.8 入大学前来自: A.城市 B.农村

**2.健康相关行为因素**

2.1 是否吸烟： A.否 B.是

*(注释：吸烟者是指每天至少吸烟一支且超过六个月或戒烟不足六个月的人)*

2.2 是否饮酒： A.否 B.是

*(注释：饮酒者是指每周至少饮酒一次且超过六个月的人)*

2.3 您一周玩网络游戏的时间：A. ＜5小时 B. ≥5小时

**3.家庭相关因素**

3.1 家庭年总收入：

A. ＜5万元 B. ≥5万元

3.2 父亲文化程度：

A.小学及以下 B.初中/高中/职高/中专 C. 大专/本科 D. 硕士及以上

3.3 母亲文化程度：

A.小学及以下 B.初中/高中/职高/中专 C. 大专/本科 D. 硕士及以上

3.4 父亲职业：

A. 教师 B. 医务人员 C. 公务员 D. 农民 E. 工人 F. 其他

3.5 母亲职业：

A. 教师 B. 医务人员 C. 公务员 D. 农民 E. 工人 F. 其他
