# Supplementary material for: Survey of health literacy level and related influencing factors in military college students in Chongqing, China: A cross-sectional analysis
Source: PLoS One. 2017 May 17;12(5):e0177776. doi: 10.1371/journal.pone.0177776 (PMC5435342; doi:10.1371/journal.pone.0177776)
Supplement: S3 File — (DOC) [file pone.0177776.s003.doc]

**The Scale of General Status of Military College Students**

**1. Sociodemographic characteristics**

1.1 **Gender**： A. male B. Female

1.2 **Age**： years

1.3 **Ethnicity**： A. Han B. other

1.4 **College**： ；

1.5 **Major**：A. medicial profession B. Non-medicial profession

1.6 **Years in college**: A.Junior（Grade one and Grade two） B. Senior（Grade three and Grade four）

1.7 **Educational system**: A. Specialist B. Undergraduate

1.8 **Residence**: A. Urban B. Rural

**2. Health-related behaviors**

2.1 do you smoke： A. Non-smoking B. Smoking

*(**NOTE：a smoker was defined as someone who had smoked at least one cigarette per day for more than six months or quitting less than six months prior to recruitment)*

2.2 do you **use alcohol***：* A. Has not used B. Has used

*(NOTE：a drinker was defined as someone who drinks alcohol at least once a week for more than six months)*

2.3 **Time playing online games**：A. ＜5 Hours B. ≥5 Hours

**3. Family-related factors**

3.1 **Annual household income**：

A. ＜ 50,000 yuan B. ≥ 50,000 yuan

3.2 **your father's education level**：

A. Primary and less B. High school C. University D. Postgraduate

3.3 **your mother's education level**：

A. Primary and less B. High school C. University D. Postgraduate

3.4 **your father's occupation**：

A. Teacher B. Medical staff C. Civil servant D. Farmer E. Worker F. Other

3.5 **your mother's occupation**：

A. Teacher B. Medical staff C. Civil servant D. Farmer E. Worker F. Other
